# Supplementary figures and images for: Polycystic ovarian syndrome (PCOS) and recurrent spontaneous abortion (RSA) are associated with the PI3K-AKT pathway activation
Source: PeerJ. 2024 Sep 6;12:e17950. doi: 10.7717/peerj.17950 (PMC11382649; doi:10.7717/peerj.17950)

A

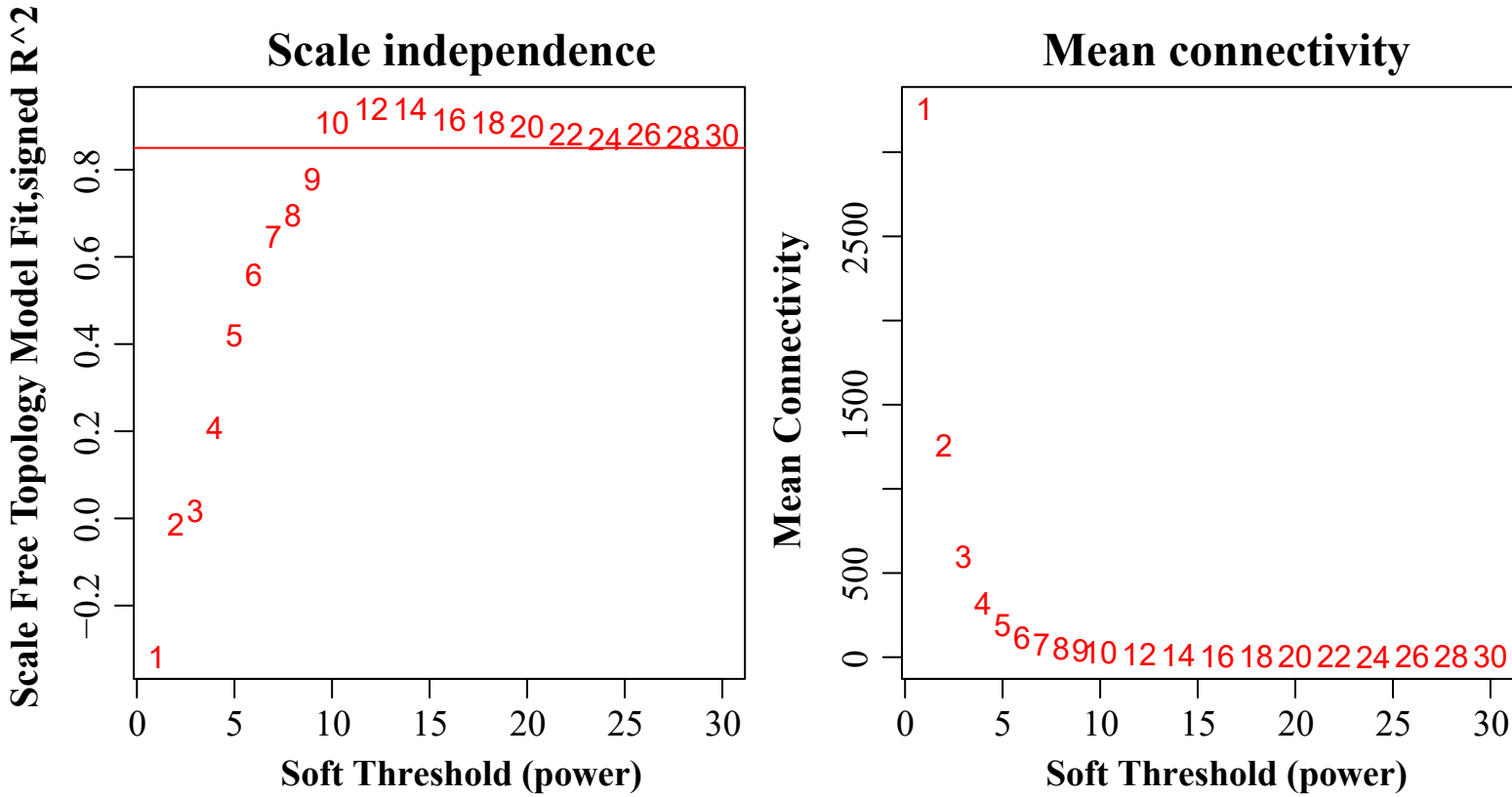

B

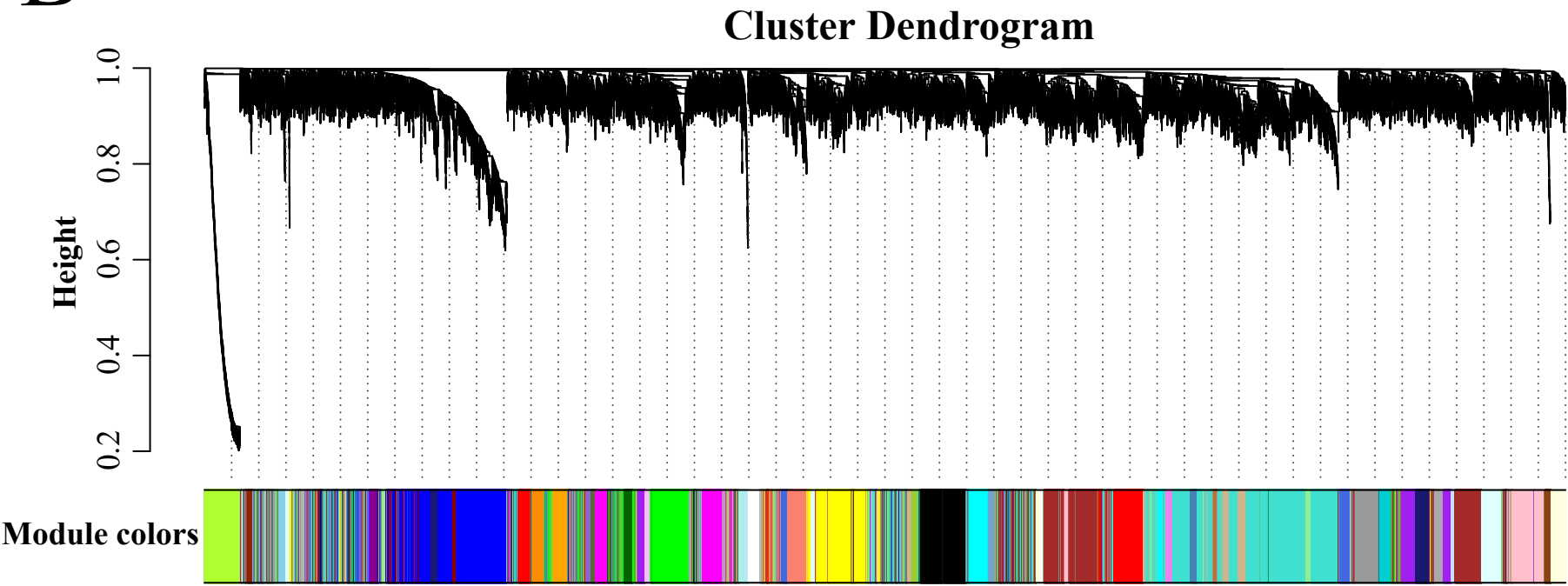

C

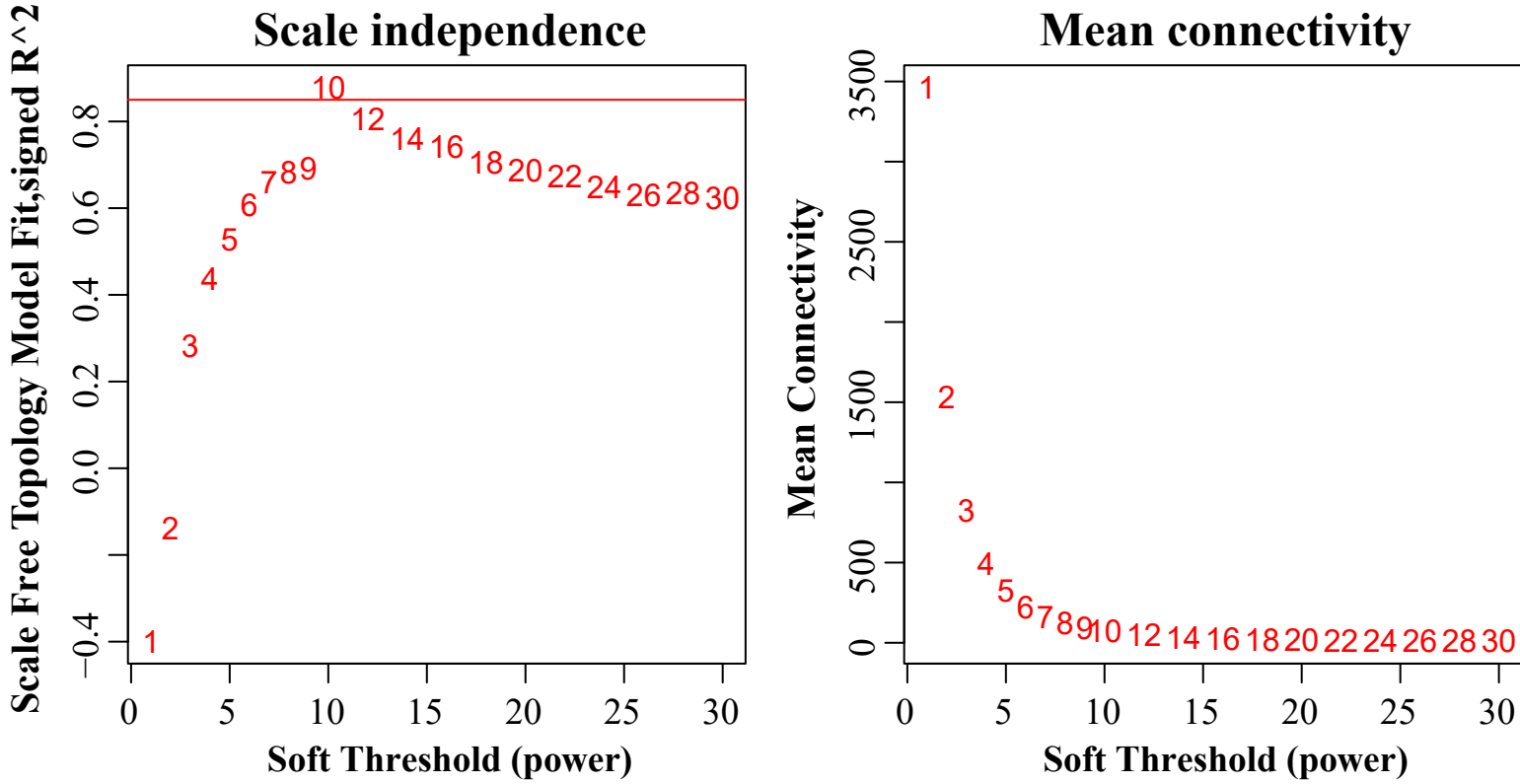

D

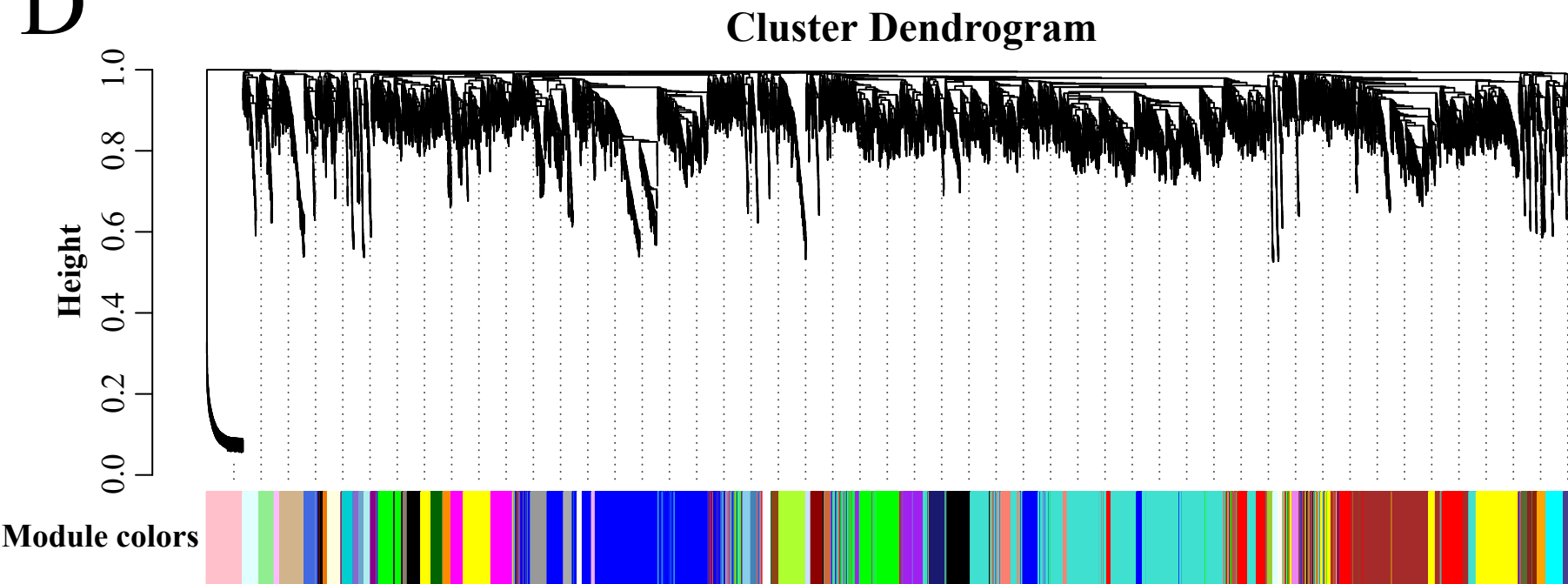

Supplement: Supplemental Information 1 — (A) Soft threshold filtering in PCOS data set and the relationship between soft threshold and connectivity. (B) Hierarchical clustering trees in WGCNA analysis within PCOS dataset. (C) Soft threshold filtering in RSA data set and the relationship between soft threshold and connectivity. (D) Hierarchical clustering tree for WGCNA analysis in RSA dataset. [file peerj-12-17950-s001.pdf]

**A**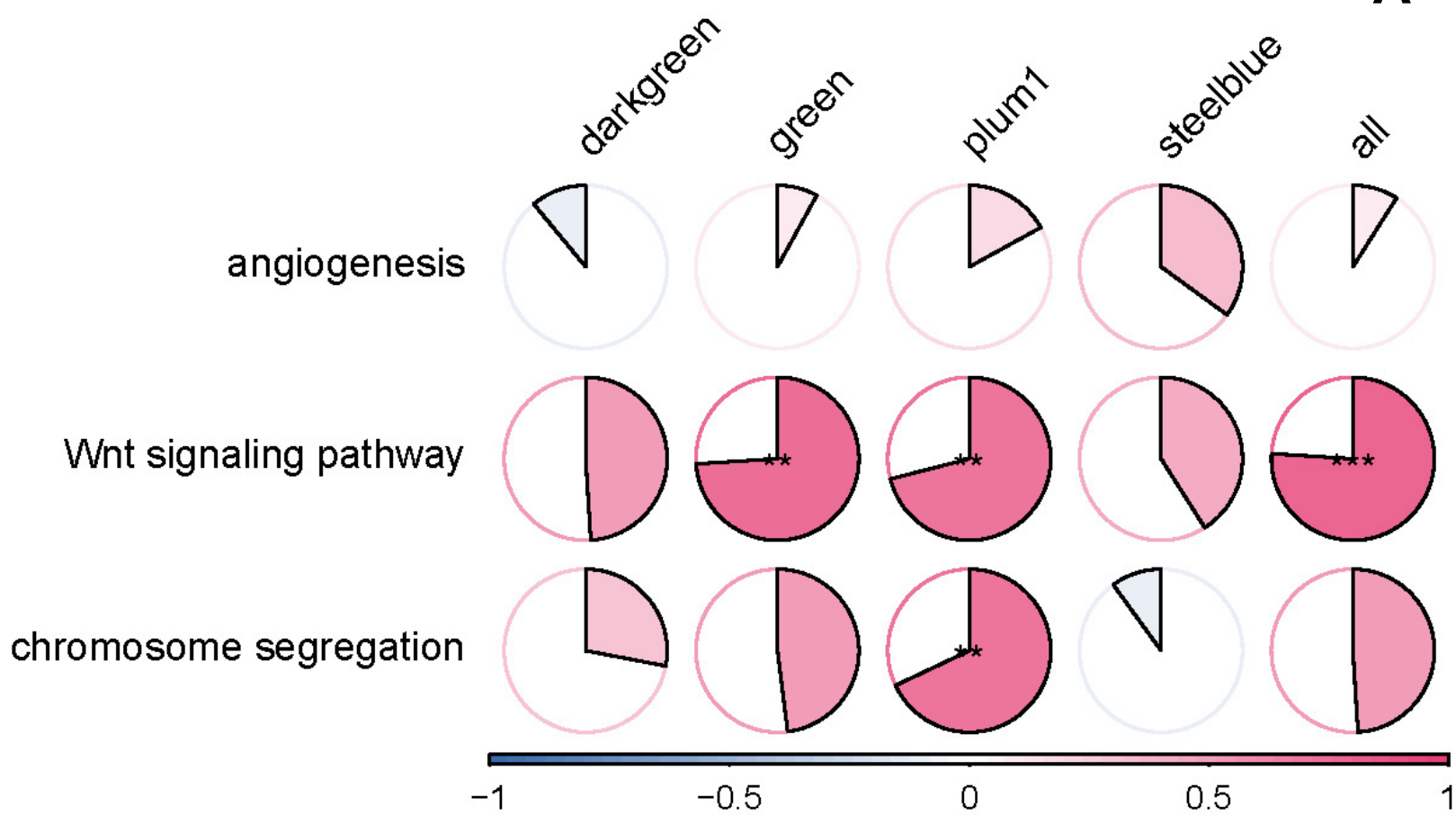**B**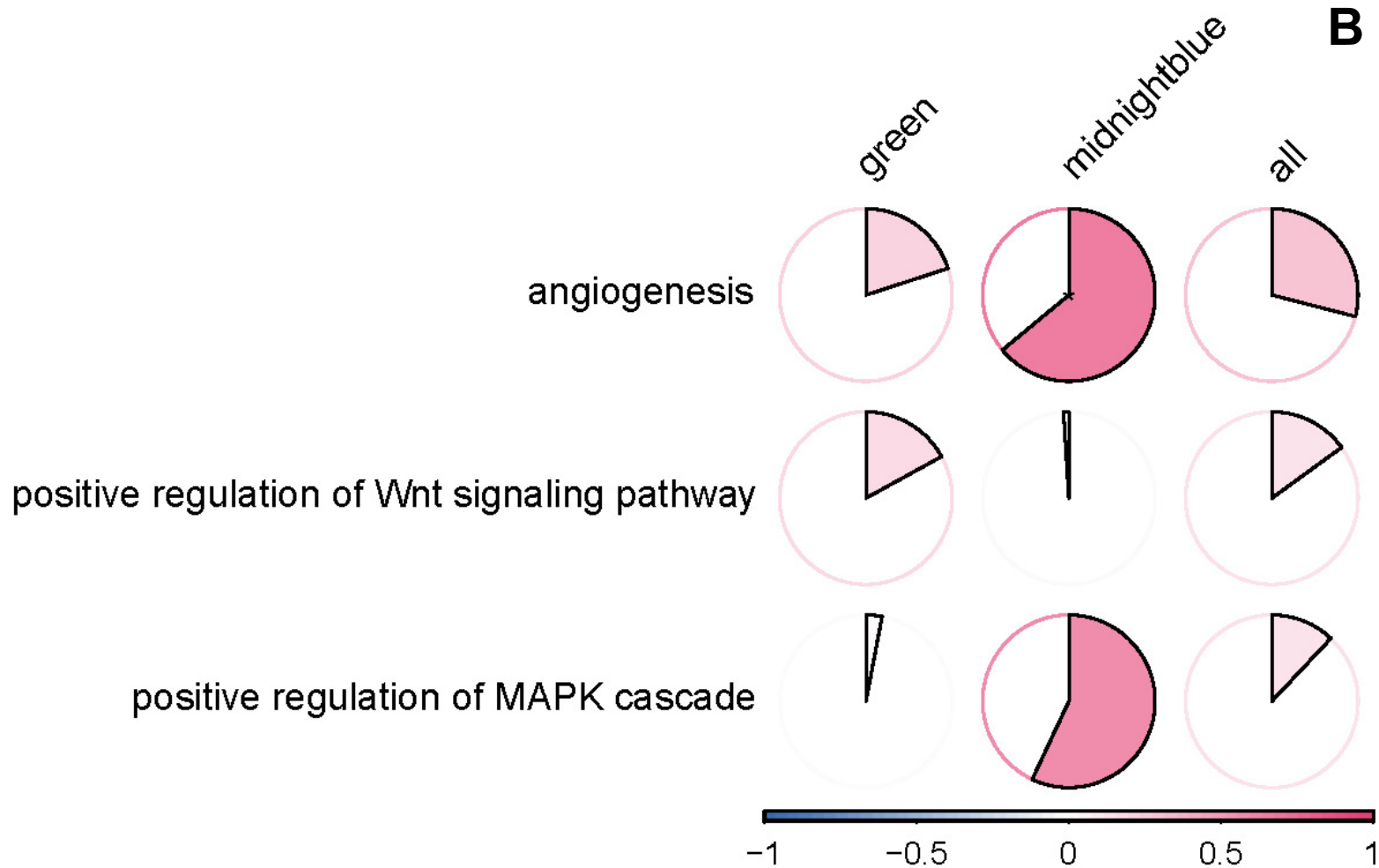

Supplement: Supplemental Information 2 — (A) Correlation analysis of gene modules significantly positively associated with PCOS (MEplum1, MEgreen, MEsteelblue, and MEdarkgreen) with angiogenesis, WNT signaling pathway, and chromosome segregation. (B) Correlation analysis of gene modules significantly positively associated with RSA (MEmidnightblue and MEgreen) with angiogenesis, WNT signaling pathway, and positive regulation of MAPK cascade. [file peerj-12-17950-s002.pdf]
